# Exploring the placement of gambling problems within the Hierarchical Taxonomy of Psychopathology

**DOI:** 10.1371/journal.pone.0313532

**Published:** 2025-03-05

**Authors:** Carla Martí Valls, Anders Håkansson, Matti Cervin

**Affiliations:** Department of Clinical Sciences, Psychiatry, Lund University, Lund, Sweden; Kyoto University Graduate School of Informatics: Kyoto Daigaku Daigakuin Johogaku Kenkyuka, JAPAN

## Abstract

The placement of gambling problems within the Hierarchical Taxonomy of Psychopathology (HiTOP) framework, which organizes psychopathology alongside broad overarching symptom spectra, is unclear. With the objective to identify associations between gambling problems and the internalizing, externalizing, and thought disorder spectra of the HiTOP, we distributed an online survey to a sample of 1005 Swedish gamblers (52.4% men, aged 18 to 60 and older). Gambling problems were measured using the Problem Gambling Severity Index, and the main HiTOP spectra were assessed with brief versions of the Inventory of Depression and Anxiety Symptoms II, the Externalizing Spectrum Inventory, and the Thought Disorder Scale. Exploratory and confirmatory factor analysis showed that the brief HiTOP scales adequately captured the internalizing, externalizing, and thought disorder spectra. Within this structure, gambling problems emerged as a distinct factor significantly correlated with all three spectra and with unique associations with each: externalizing (β =  0.33, *p* < .001), thought disorder (β =  0.30, *p* = .001) and internalizing (β =  0.22, *p* = .022). In men, gambling problems were significantly associated with the thought disorder (β =  0.54, *p* < .001) and externalizing (β =  0.31, *p* < .001) spectra. In women, gambling problems were significantly associated with the externalizing (β =  0.39, *p* < .001) and internalizing (β =  0.35, *p* = .013) spectra. Our study is a first attempt to link gambling problems to the three main spectra of the HiTOP. Our findings show that gambling problems are associated with all three spectra and that there may be potential gender differences in the associations between gambling problems and co-occurring psychopathology.

## Introduction

Gambling refers to the act of risking something of value on an uncertain outcome to win more. Between 30% and 90% of adults have gambled at least once and 1-3% show gambling patterns that may be considered at risk of or being pathological [1,2]. Popular forms of gambling include lotteries, sports betting, and casino games, with online gambling being on the rise [3,4]. In Sweden, over half the population gambled in 2022, with 4% being at risk of gambling problems and 0.5% experiencing pathological gambling [4]. In the Swedish context, gambling has also increasingly become overwhelmingly online-based in recent years, with nearly all individuals seeking treatment reporting that they gamble online. For example, a recent study reported rates of 66% of online casino gambling and 26% of sports betting among treatment-seeking clients [5]. Likewise, online casino gambling is strongly predominating in televised gambling advertisements [6].

Co-occurring psychopathology is common among individuals with gambling problems. Rates for substance use disorders (SUDs) range from 22% to 58%, with an estimated lifetime prevalence of 47% [7–9]. Mood disorders (23.1% to 37.9%), personality disorders (28.8%), and anxiety disorders (17.6% to 37.4%) are also prevalent [8–10]. However, most research assessing co-occurring psychopathology in gambling problems has relied on binary classifications for both gambling problems and mental disorders (i.e., that they are either absent or present), as suggested by the *Diagnostic and Statistical Manual of Mental Disorders* (5th ed.; DSM–5) [7]. This contrasts emerging evidence suggesting that psychopathology is better conceptualized along interrelated dimensional spectra, and that thresholds between normal and abnormal are arbitrary [11,12]. Quantitative empirical alternatives to binary classification models are emerging, with a prominent model being the Hierarchical Taxonomy of Psychopathology (HiTOP) [12].

The HiTOP is a quantitative dimensional framework of psychopathology where psychopathological phenomena are classified hierarchically into different levels of specificity based on covariance of symptoms [12]. The levels of the HiTOP range from specific signs, symptoms, and behaviors (e.g., suicidality, impulsivity), to integrated symptom components (e.g., agoraphobia, bipolar disorder), dimensional syndromes (e.g., externalizing, internalizing, and thought disorder symptoms), and finally, a general psychopathology factor (often referred to as the p factor), which represents general vulnerability towards all mental health problems [11,12]. The main spectra within the HiTOP are the internalizing, externalizing, and thought disorder spectra. The internalizing spectrum is characterized by excessive negative emotions like fear, distress, and anhedonia. Externalizing symptoms refer to outward behavioral issues, including aggression, manipulation, impulsivity, and risk-taking. The thought disorder spectrum encompasses odd thoughts and perceptions such as delusions, hallucinations, and magical thinking [12]. Two additional spectra have been proposed: the detachment spectrum, marked by social withdrawal, apathy, or lack of emotional expression, and the somatoform spectrum, defined by difficulties without any underlying medical cause, such as somatization [12]. At current, the placement of gambling problems within the HiTOP is unclear. Some research suggests that pathological gambling may be most strongly linked to the externalizing spectrum, but that this association may be weaker compared to other externalizing difficulties, such as SUDs [13–15]. For instance, using factor analysis, Oleski and colleagues [14] showed that pathological gambling loaded most strongly onto the externalizing factor (standardized factor loading: 0.49), but with a weaker loading than alcohol dependence (0.74), antisocial personality (0.74) and substance dependence (0.89). Similarly, Milosevic [16] found that the externalizing factor accounted for most of the variance among gambling problems (31%) and that a secondary internalizing factor accounted for additional variance (16%). King and colleagues [13] analyzed gambling problems in a sample of 1,329 young adult twins at the age of 25 and their factor analyses revealed that gambling problems only loaded 0.31 onto the externalizing factor, which was much lower than antisocial behavior (0.72), addictions to nicotine (0.57), alcohol (0.56), and cannabis (0.42). Likewise, Richard and colleagues [15] found even lower loadings of adolescent gambling (0.18) onto the externalizing spectrum, again, lower than gaming (0.32) and gambling frequency (0.28), and much lower than a range of typical externalizing behaviors like drug use, rule-breaking, and fighting (0.45-0.76). Potential gender differences in how gambling problems are linked to co-occurring psychopathology have also been suggested. In Oleski and colleagues [14], a significant association between gambling problems and the Anxious-Misery factor, corresponding to the distress factor in the HiTOP [12], was only observed in women. Other studies have found no gender differences [13,15]. Notably, no study has examined the association between gambling problems and the thought disorder spectrum. However, dissociation, a key component of the thought disorder spectrum, has been linked to gambling disorder in previous research [17].

The objective of the present study was to examine how gambling problems are associated with the broad internalizing, externalizing, and thought disorder HiTOP spectra. Based on the aforementioned findings, we hypothesized that gambling problems would be most clearly associated with the externalizing spectrum, and significantly but less strongly associated with the internalizing spectrum. Potential associations between gambling problems and the thought disorder spectrum, as well as gender differences in the associations between gambling problems and the HiTOP spectra, were examined without pre-specified hypotheses.

## Materials and methods

### Sample

Participants were recruited from August 16 to October 12, 2023, via a web survey conducted by the market research company Ipsos. Eligible individuals were aged 18 or older, had gambled for money at least once in the past 12 months, and lived in Sweden. Participants (*N* =  1005; 52.4% men) lived in various regions of Sweden, predominantly Stockholm (24.9%), Västra Götaland (17.6%), and Skåne (13.1%). They were evenly distributed across age groups: 18-29 (20.0%), 30-39 (23.1%), 40-49 (18.4%), 50-59 (15.1%), and 60 and older (23.4%). Most were employed (68.2%) and held a university (48%) or high school (41.8%) diploma. The majority reported gambling 10 or more times in the last year (69.3%), primarily online, including sport betting (67.9%), horse racing betting (50.6%), and casino games (47.8%). A small percentage self-reported previous diagnoses of gambling disorder (6.3%), alcohol/substance use disorder (7.7%), and/or other psychiatric disorders (22%). Extended demographic information is in S1–S3 Tables in the online supporting information.

### Scale translation

Permission to use and translate the Externalizing Spectrum Inventory [18] and the Thought Disorder scale [19] was granted by the original authors in November 2022. Swedish translations followed the forward-backward translation model, with forward translations by two of the authors (M. C. and A. H.) in January 2023, and back-translation by a bilingual doctoral student from Malmö’s Clinical Addiction Research Unit. The final questionnaires were carefully reviewed to ensure accuracy, completed by February 2023. Refer to the final scale translated into Swedish in the online supporting information.

### Procedure and ethical approval

The study aimed to recruit 1000 participants, with the final sample size being determined by availability. After excluding participants with missing data or incomplete responses (35.2%), Ipsos, the web-survey market company in charge of the data collection, provided the finalized dataset. Written consent was obtained electronically, and participants received credits from Ipsos upon completing the survey. The study was approved by the Swedish Ethical Review Authority (file number 2023-00802-01, approval date: March 8, 2023). The study collected sensitive personal data related to health, along with demographic information such as gender, age, education level, and employment status. The health data primarily consisted of detailed questions about gambling patterns and potential gambling problems, as well as questions regarding diagnoses related to gambling, other addictions, and psychological symptoms across various psychiatric domains. Data were collected anonymously from the Ipsos web panel, ensuring that no information could directly or indirectly identify individuals. Additionally, Ipsos does not have access to individual responses, which protects the integrity of the research participants.

### Instruments

#### Problematic gambling.

Problematic gambling behaviors were assessed using the Swedish version of the Problem Gambling Severity Index (PGSI) [20,21]. The PGSI is a nine-item scale that evaluates the severity of gambling-related problems in the general population over the past 12 months, covering information about financial, social, and psychological consequences of gambling. Each item is rated on a 4-point Likert scale (0 = never to 3 = almost always). The total score ranges from 0 to 27, with the cutoffs of 0 for non-problematic behavior, 1 to 2 for low problems, 3 to 7 for moderate problems, and 8 or higher for severe problems [20]. In the present sample, the scale demonstrated high internal consistency (Cronbach’s alpha; α = .95, McDonald’s Omega; ω = .96).

#### Co-occurring psychopathology.

Co-occurring psychopathology was assessed using subscales from the most up-to-date HiTOP measures: the Inventory of Depression and Anxiety Symptoms II (IDAS-II) [22], the Externalizing Spectrum Inventory (ESI) [18] and the Thought Disorder Scale (TD) [19], covering the three major overarching spectra of the HiTOP. The IDAS-II is a self-report inventory with 99 items rated on a 5-point Likert scale (1 = Not at all to 5 = Extremely). It assesses a broad range of symptoms of the internalizing spectrum in the HiTOP, mainly connected to anxiety and mood disorders, with three overarching factors: Distress, OCD and Positive Mood [22]. The ESI is a 100-item self-reported inventory where each item is rated using a 4-point Likert scale (1 = True to 4 = False). It measures problematic behaviors and traits connected to the externalizing spectrum of the HiTOP with three overarching factors: Disinhibition, Callous-Aggression, and Substance Abuse [23]. Finally, the TD is a self-report questionnaire with 215 items, each rated on a 4-point Likert scale (0 = Not at all to 3 = A lot), that captures the thought disorder spectrum of the HiTOP. It is based on positive and negative or disorganized traits/symptoms of schizophrenia-related disorders [19]. For each scale, higher scores indicate more severe symptoms. To minimize participation burden, we reduced the number of items of each scale. To promote consistency and ensure psychometric equivalence, we limited the number of subscales per overarching factor to three. Our selection criteria were based on previous psychometric evaluations. We reviewed overarching factor structures and inspected the factor loadings from previous studies to identify subscales with strong loadings onto the main factor, while also ensuring that they represented the full breadth of each spectrum.

For the IDAS-II, considering both the original study and the Swedish validation of the scale, Dysphoria was the subscale that loaded most strongly onto the broad Distress factor [22,24]. Dysphoria also captures the core symptoms of depression and anxiety, addressing the broad internalizing spectrum more comprehensively than the other subscales, which focus on more specific aspects like panic or traumatic intrusions [24]. In contrast, the Cleaning, Ordering, and Checking subscales all loaded clearly onto the OCD factor [22,24]. As a result, we incorporated these subscales into our measure as indicators of the broad internalizing spectrum. For length purposes, the OCD subscales were reduced to three items each, using item factor loadings from the adult sample from the Swedish validation study [24]. We followed the same procedure regarding the ESI. Based on the original studies, the subscales of Alcohol Problems, Drug Problems, and Marijuana Problems loaded most strongly onto the Substance Abuse factor; and Irresponsibility, Problematic Impulsivity, and Theft subscales onto the Disinhibition factor [18,23]. Due to their broad liability for externalizing spectrum disorders [25], Disinhibition and Substance Abuse factors were retained, while the more specific Callous-Aggression factor was excluded. As the TD questionnaire is still in the preliminary stage of scale reduction, we could only include one subscale due to its extensive length. We selected the Dissociation subscale due to its robust loadings onto the overarching thought disorder spectrum both in a general and a clinical sample [19]. Thus, our final measure of the HiTOP spectra comprised 71 items: 36 from ESI (15 from Substance Abuse, 21 from Disinhibition), 16 from TD (all from Dissociation), and 19 from IDAS-II (10 from Dysphoria, 9 from OCD). The original response scales were preserved, and the subscales presented good to excellent internal consistency in the present sample: Dysphoria (α = .94; ω = .95), Cleaning (α = .88; ω = .89), Ordering (α = .86; ω = .87), Checking (α = .89; ω = .89), Alcohol Problems (α = .87; ω = .88), Drug Problems (α = .95; ω = .97), Marijuana Problems (α = .93; ω = .95), Irresponsibility (α = .90; ω = .93), Problematic Impulsivity (α = .92; ω = .94), Theft (α = .83; ω = .88), and Dissociation (α = .97; ω = .98).

### Data analysis

R Studio version 4.3.2 [26] was utilized for data analysis, including descriptive statistics and Spearman’s correlations for all study variables. Cronbach’s alpha (α) and McDonald’s omega (ω) were calculated for each scale. First, we modeled the items selected from existing HiTOP scales alongside the gambling items to find an empirically justified HiTOP model. To ensure that the reduced items still represented the original constructs of the scales, we conducted an exploratory factor analysis (EFA). The sample was randomly split 1:1, and we used the first half (*n* =  502) for EFA. As the items were ordinal, a correlation matrix was computed using polychoric correlations. Two diagnostic tests, the KMO test for sampling adequacy (ideal common variance >  0.80) and Bartlett’s test of sphericity (significant p-value indicating variable interdependence), assessed the data’s suitability for EFA. Parallel analysis determined the number of factors to retain (*psych* package with principal axis factoring and *promax* rotation). The final model was then evaluated using confirmatory factor analysis (CFA) in the second half of the sample (*n* =  503) (*lavaan* package) [27]. Diagonally Weighted Least Squares (DWLS) estimation was used for modeling the non-normal ordinal item data in CFA (Mardia’s coefficient of multivariate normality: *p* < .001) [28]. Model fit was evaluated using fit indices: RMSEA <  0.06, SRMR <  0.09, TLI >  0.90-0.95, and CFI >  0.90-0.95, adhering to Hu and Bentler [29] recommendations. Chi-square difference tests compared nested models, with significance indicating better fit for the more complex model, calculated using Satorra–Bentler chi-square correction (S - χ^2^) for non-normally distributed data [30]. In accordance with the literature, we tested two different higher-order factor solutions: OCD placed under the internalizing spectrum [12] and OCD placed under the thought disorder spectrum [11]. A hierarchical model representing an overarching *p* factor was also tested for the best-fitting model. Reliability of latent factors was evaluated using Cronbach’s alpha (α) and McDonald’s omega (ω). Gender invariance was assessed via multigroup CFA, constraining model parameters across three levels: configural, scalar, and strict invariance. Invariance was indicated by a ΔCFI < .01 [31]. Two participants from other gender categories (other/unsure) were excluded due to low statistical power, leaving final samples of 527 men and 476 women.

To examine associations between gambling problems and the HiTOP spectra, we first analyzed covariance coefficients between gambling problems and the overarching HiTOP spectra using the full sample. Then, to identify unique associations, we ran a path model where the HiTOP spectra were used as predictors of gambling problems. Although the latter model implies causality, it efficiently reveals unique associations between HiTOP spectra and gambling problems, paving the way for future confirmatory research. Thirteen outliers showing high dissociation but low co-occurring psychopathology, including gambling problems, were excluded due to their impact on linearity, which was confirmed through bivariate scatterplots using latent factor scores. Gender differences in the association between gambling problems and HiTOP spectra were examined using separate SEM models for men (*n* =  518) and women (*n* =  472). Multicollinearity was assessed by fitting a linear model of the HiTOP spectra predicting the gambling problems factor, all derived from factor scores, using the variance inflation factor (VIF) (VIF <  3 (ideal), 3-5 (acceptable), >  10 (problematic)) [32]. Statistical significance was established at *p* < .05. The R code used for the analyses can be publicly accessed at: https://osf.io/8sa25/.

## Results

Descriptive statistics are presented in Table 1. Utilizing the PGSI threshold [20], 56.4% of the sample exhibited no risk of developing gambling problems, 17.2% displayed a low risk, 13.2% a high risk, and 13.1% manifested gambling problems. Spearman correlations between all symptom scales were highly statistically significant (*p* < .001) and exhibited no multicollinearity (*r* ≤ .60). The correlations between the scales were the following: IDAS-II and ESI (*r* = .60), IDAS-II and TD (*r* = .60), ESI and TD (*r* = .59), ESI and PGSI (*r* = .52), IDAS-II and PGSI (*r* = .48) and PGSI and TD (*r* = .44).

**Table 1:** Descriptive statistics for the total sample and stratified by gender.

Scales	Total (*N* = 1005)		Women (*n* = 476)		Men (*n* = 527)	
	*M*	*Mdn*	*M*	*Mdn*	*M*	*Mdn*
	*(SD)*	*(Q1-Q3)*	*(SD)*	*(Q1-Q3)*	*(SD)*	*(Q1-Q3)*
PGSI	2.51	0	3.06	0	2.01	0
	(4.6)	(0-3)	(5.3)	(0-4)	(3.8)	(0-2)
ESI	45.62	38	46.79	38	44.53	38
	(16.9)	(36-46)	(17.8)	(36-50)	(15.9)	(36-44)
IDAS-II	31.51	26	34.76	29	28.55	25
	(13.6)	(21-37)	(15.4)	(22-44)	(11.1)	(21-32)
TD	4.12	0	4.66	0	3.62	0
	(8.8)	(0-3)	(8.9)	(0-5)	(8.7)	(0-2)

Two participants missing across gender groups (1 for “Other” and 1 for “Unsure”).

M: Mean; SD: Standard Deviation; Mdn: Median; Q1-Q3: First and third quartiles.

PGSI: Problem Gambling Severity Index [20]; ESI: Externalizing Spectrum Inventory [18]; IDAS-II: Inventory of Depression and Anxiety Symptoms II [22]; TD: Thought Disorder Scale [19].

To identify an adequate HiTOP model, we conducted an EFA on the first part of the sample (see the polychoric matrix in the online supporting information). Diagnostic tests for EFA suitability yielded positive results, with an overall KMO test value of 0.93 and item-level KMO ranging from 0.86 to 0.98. Additionally, Bartlett’s test of sphericity was highly significant (*p* < .001). Parallel analysis suggested 7 factors. We compared different factor solutions (from 7 to 1 factor), considering cross-loadings, correlations among factors, total variance explained, and parsimony. A 5-factor solution was most parsimonious, accounting for 75% of the variance. In contrast, the 7-factor solution yielded a factor with residual variance and no primary item loadings, while both the 6- and 4-factor solutions presented a lower variance explained (74% and 72% respectively). Item loadings of the final 5-factor solution and closer solutions (7- and 6-factor) can be found in S4–S6 Tables in the online supporting information. The latent factors in the 5-factor model were: Externalizing, Thought Disorder, Dysphoria, Gambling problems, and OCD.

After establishing the measurement model, we fitted this model using the other half of the sample, specifying all derived factors from the EFA except for gambling problems (model 1). Gambling problems were omitted because we were interested in finding the overarching structure among the traditional HiTOP spectra and then link the most parsimonious spectra to gambling problems. The model presented a good fit (see Table 2) and all latent factors showed high internal consistency: Externalizing (α = .98; ω= 1.02), Thought Disorder (α = .98; ω=.99), OCD (α = .94; ω=.99) and Dysphoria (α = .94; ω=.98). The model was compared to two similar models incorporating higher-order factors according to the literature: one with OCD placed under the internalizing spectrum (model 2) and the other with OCD under the thought disorder spectrum (model 3). All models demonstrated good fit (refer to Table 2). Model comparison between the two best models (1 and 2) revealed that adding complexity significantly improved the model fit (p-value of χ² difference test < .05), suggesting that model 2 best represented the data. Additionally, we fitted a hierarchical version of model 2, aligning with the HiTOP literature regarding a general psychopathological factor (p factor) (refer to Table 2). However, the addition of this factor did not significantly improve its fit (p-value of χ² difference test > .05); therefore, model 2 was retained. Standardized item loadings of the final model (model 2) are available in S7 Table in the online supporting information. We tested measurement invariance across gender using the same model (model 2) with the entire sample (*n* =  1003; 527 men and 476 women). All invariance models demonstrated good fit, and invariance held across both groups (ΔCFI =  0.004), indicating that the factor structure, factor loadings, intercepts, and residual variances of the latent constructs were statistically equivalent in both genders. For detailed fit indices, please refer to model 5 in Table 2.

**Table 2:** Model fit statistics.

Models		*n*	*S* *-* χ*2*	*df*	*p*	CFIs	TLIs	RMSEAs	SRMR
1	OCD as its own factor^a^	503	3919.696	2408	<.001	0.975	0.974	0.035	0.061
3	OCD and dissociation under TD	503	5114.133	2402	<.001	0.955	0.953	0.047	0.080
2	**OCD and dysphoria under INT**	503	3917.627	2409	<.001	0.975	0.974	0.035	0.061
4	Hierarchical version	503	6520.634	3072	<.001	0.973	0.972	0.033	0.053
5	Gender invariance								
	Configural	1003	9061.403	6140	<.001	0.978	0.977	0.031	0.060
	Scalar	1003	8680.689	6314	<.001	0.982	0.982	0.027	0.064
	Strict	1003	8775.635	6394	<.001	0.982	0.982	0.027	0.064
6	Gambling problems regressed on model 2	992	6578.095	3072	<.001	0.971	0.971	0.034	0.053
7	Men	518	4106.613	3072	<.001	0.984	0.983	0.026	0.053
8	Women	472	5007.880	3072	<.001	0.969	0.968	0.037	0.067

All fit indices except SRMR are scaled (s).

^S^- χ2: Satorra–Bentler chi-square correction for non-normally distributed data; df: Degrees of freedom; p: p-value; CFI: Comparative Fit Index; TLI: Tucker-Lewis Index; RMSEA: Root Mean Square Error of Approximation; SRMR: Standardized Root Mean Square Residual.

INT: Internalizing; EXT: Externalizing; TD: Thought Disorder.

^a^As suggested by the EFA. In **bold,** the best model according to the chi-square difference test.

**Fig 1:**
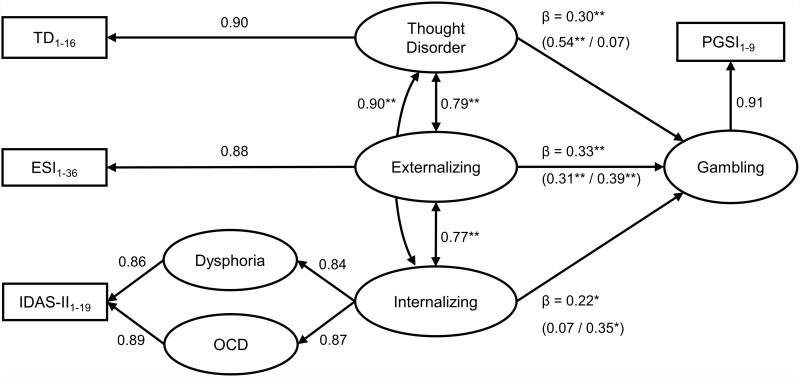
Final model (model 2) with gambling problems regressed (model 6). Loadings and beta coefficients are standardized, and item loadings under each factor are averaged for simplicity. All items significantly loaded into their respective factors (*p* ≤ .001). Significant path relationships are indicated with ** for *p* ≤ .001 and *  for *p* < .05. Regression totals include separate values for men and women in parentheses, respectively. PGSI: Problem Gambling Severity Index [20], ESI: Externalizing Spectrum Inventory [18], IDAS-II: Inventory of Depression and Anxiety Symptoms II [22], TD: Thought Disorder Scale [19].

Finally, to evaluate the association between gambling problems and the broad HiTOP spectra, we added gambling problems to model 2 and evaluated the standardized covariance coefficients between gambling problems and the externalizing, internalizing, and thought disorder spectra. Results showed that gambling problems were similarly correlated to the thought disorder (*r* =  0.76), internalizing (*r* =  0.75), and externalizing (*r* =  0.74) spectra. To parse out unique associations, we added a regression path from the HiTOP latent factors to the latent factor of gambling problems. The model demonstrated good fit (model 6 in Table 2). Regression paths revealed that gambling problems were primarily associated with the Externalizing (β =  0.33, SE =  0.05, *p* < .001) and Thought Disorder (β =  0.30, SE =  0.09, *p* = .001) factors, followed by the Internalizing factor (β =  0.22, SE =  0.13, *p* = .022). No multicollinearity problems were detected across the latent factors (VIF =  2.7 to 3.3). We also investigated structural differences across genders by conducting two separate CFAs in the entire sample. Both models showed good fit (refer to models 7 and 8 in Table 2). Standardized item loadings for both models can be found in S7 Table. Significant differences emerged across gender groups. In men, gambling problems were significantly associated with the Thought Disorder (β =  0.54, SE =  0.12, *p* < .001) and Externalizing (β =  0.31, SE =  0.07, *p* < .001) factors, while the Internalizing factor remained non-significant (*p* > .05). In women, gambling problems were significantly associated with the Externalizing (β =  0.39, SE =  0.08, *p* < .001) and Internalizing (β =  0.35, SE =  0.17, *p* = .013) factors, while the Thought Disorder factor remained non-significant (*p* > .05). For further information on the regression coefficients please refer to S8 Table in the online supporting information. For a visual representation of the final model see Fig 1.

## Discussion

The present study aimed to examine how gambling problems are associated with the overarching spectra of the HiTOP model. To our knowledge, this is the first study to include the thought disorder spectrum, and to use subscales from the IDAS-II [22], the ESI [18], and the TD [19] for assessing co-occurring symptoms in this context. The results revealed that gambling problems emerged as a distinct factor that showed significant and strong correlations with all HiTOP spectra. When analyzing more unique associations, gambling problems were most strongly associated with the externalizing spectrum, followed by thought disorder spectrum, and to a lesser extent, the internalizing spectrum. Further, gender differences were observed in the association between gambling problems and the main HiTOP spectra. In men, gambling problems were most clearly associated with the thought disorder spectrum followed by the externalizing spectrum, while in women, they were most strongly associated with the externalizing spectrum, followed by the internalizing spectrum.

Our results highlight three main findings. First, they show that gambling problems are strongly associated with all the major forms of psychopathology included in the HiTOP. Second, they confirm the unique link between gambling problems and externalizing symptoms [13–15], supported by the well-established association between gambling problems, SUDs and impulsivity [7–10]. Third, they reveal a significant difference in how gambling problems are associated with both the internalizing and thought disorder spectra across genders. Notably, the association between gambling problems and the thought disorder spectrum observed in men is a novel finding. While dissociation has previously been linked to gambling disorder, gender differences in this association have not been previously examined [17]. Possible reasons for this association could be related to the novel assessment of gambling problems in connection with the thought disorder spectrum within the HiTOP framework, as well as to specific sample characteristics stemming from the recruitment methods used. To further understand the link between gambling problems and the thought disorder spectrum, additional research is needed. Conversely, the association between gambling problems and the internalizing spectrum in women has been previously reported. For instance, Oleski and colleagues [14] found a significant path between pathological gambling and the Anxious-Misery factor only in women [14,16]. This secondary path to gambling in women has been hypothesized to be connected to a higher prevalence of mood disorders and emotion regulation problems, and their impact on gambling behaviors [14].

The study’s large and diverse sample strengthens generalizability, but online recruitment may skew towards frequent internet users, potentially limiting applicability, especially for severe gambling cases and diverse contexts. The study is also limited by the selected measurement tools. We only used a minority of the subscales commonly used to assess the HiTOP spectra. Likewise, the tool for assessing gambling problems primarily focuses on behavior rather than symptoms and traits. While the present findings are consistent with prior research, our results also suggest that the placement of gambling problems within the HiTOP framework is not straightforward, as gambling problems relate to all spectra, and gender differences should be acknowledged. To better understand the placement of gambling problems within the broader psychopathological landscape, longitudinal studies that use brief but valid measures across the major subfactors of the HiTOP are needed.
